# Association of Psoriasis With Thyroid Disorders: A Tertiary Center-Based Cross-Sectional Study

**DOI:** 10.7759/cureus.79706

**Published:** 2025-02-26

**Authors:** Mohammed A Alosaimi, Mishari T Alrubaiaan, Aser M Alsuhaymi, Saad A Alsulaiman, Abdulmalik M Alanazy, Abdullah I Al Eissa, Saleh M Alosaimi, Muath Owaidh Alilaj

**Affiliations:** 1 College of Medicine, King Saud Bin Abdulaziz University for Health Sciences, Riyadh, SAU; 2 College of Medicine, King Abdullah International Medical Research Center, Riyadh, SAU; 3 Family Medicine and Primary Care Department, King Abdulaziz Medical City, Riyadh, SAU; 4 Family Medicine and Primary Care Department, King Abdullah International Medical Research Center, Riyadh, SAU

**Keywords:** anti-tg, anti-tpo, autoimmune disease, ft3, ft4, psoriasis, thyroid dysfunction, tsh

## Abstract

Background

Psoriasis is a chronic inflammatory skin disease associated with various entities. Despite indications of a correlation, this relationship remains poorly understood due to inconsistent findings in previous studies and the lack of sufficient data. This study aims to investigate thyroid dysfunction and autoimmunity prevalence in psoriasis patients at King Abdulaziz Medical City, Riyadh and explores their relationship with gender, age, and disease duration.

Methods

A retrospective cross-sectional study was conducted involving psoriasis patients diagnosed between 2015 and 2023. We included patients aged 18 or older with a confirmed psoriasis diagnosis. We excluded patients under 18, pregnant or lactating women, and those with pre-existing thyroid conditions or treatments affecting thyroid function. Statistical analyses were performed using SPSS Inc. Released 2003. SPSS for Windows, Version 12.0. Chicago, SPSS Inc., with significance set at p < 0.05.

Results

One hundred and nineteen individuals, with 41 males (34.4%) and 78 females (65.6%), were part of the study. The average age was 44.19 years. The average disease duration was 5.35 years. The most common initial sites of involvement were the extremities (35.3%), the scalp (25.2%), and the trunk (10%). The most prevalent type was chronic plaque psoriasis (74.8%), pustular (12.6%), erythrodermic (4.2%), palmoplantar (3.4%), guttate (2.5%), and unknown (2.5%). Thyroid function tests revealed that 38.7% had abnormalities, with 31.1% elevated thyroid-stimulating hormone (TSH) and 7.6% low TSH. Triiodothyronine (FT3) levels varied, with 9.2% having low FT3 and 10.9% having high FT3. Moreover, Thyroxine (FT4) also varies, with 7.6% having low FT4 and 2.5% having high FT4. Elevated antithyroglobulin antibodies (anti-TG) and thyroid peroxidase antibodies (anti-TPO) were observed in 12.6% and 16.0% of subjects, respectively.

Conclusion

The study revealed a notable prevalence of thyroid dysfunction among psoriasis patients, with no direct correlation between psoriasis types and thyroid abnormalities.

## Introduction

Psoriasis is a chronic inflammatory skin disease with a strong genetic component and autoimmune characteristics [1]. It is linked to various comorbidities like psoriatic arthritis, heart and metabolic diseases, and mental health problems, which can all negatively impact one's quality of life [1]. Globally, the estimated prevalence is around 2%. However, the prevalence of the disease varies between countries [2,3]. Different demographics and ethnic groups worldwide experience varying levels of psoriasis [2]. Within Saudi Arabia, the prevalence also differs by region over time, showing an overall increasing trend recently [2]. The northern Al-Jouf region reported the highest rate at 5.33% [2]. Estimates in eastern and southwestern Saudi Arabia were 1.5% and 3.4%, respectively [2]. The specific pathophysiological pathways that cause psoriasis to develop are very complicated [4]. It is an immune-mediated condition characterized by unusually active innate and adaptive immune system cells and molecules [4]. According to Rendon et al., 2019, immune cell function impairment is largely mediated by keratinocytes, the most prevalent form of epidermal cells [4]. In addition, psoriasis has a complicated genetic foundation due to its multifactorial nature [4].

Psoriasis and thyroid dysfunction might interplay, but the exact nature of this relationship is not fully understood. Several studies have suggested a potential link between the two disorders. Studies from India and China have found that psoriasis patients often have abnormal thyroid function, with some showing elevated levels of anti-thyroid peroxidase (anti-TPO) antibodies [5-7]. Also, psoriasis patients with thyroid dysfunction tend to have more severe forms of the disease [5]. Different types of psoriasis, such as psoriasis vulgaris and erythrodermic psoriasis, are associated with varying thyroid hormone levels, with erythrodermic psoriasis patients showing normal levels of thyroid-stimulating hormone (TSH) and lower free triiodothyronine (FT3) or free thyroxine (FT4) levels. On the other hand, patients with psoriasis vulgaris had higher levels of TSH [6]. Furthermore, one large study indicated that both psoriasis and psoriatic arthritis patients are at a higher risk for thyroid dysfunction, including hyperthyroidism and Hashimoto's thyroiditis, compared to non-psoriasis controls [8]. However, there is a gap in studies examining the prevalence and relationship of psoriasis and thyroid dysfunction in Saudi Arabia and the Gulf region. This study aims to explore the prevalence of thyroid dysfunction and autoimmunity in psoriasis patients at King Abdulaziz Medical City, as well as the relationship between these conditions and factors like gender, age, and disease duration.

## Materials and methods

Study design

Retrospective cross-sectional study at King Abdulaziz Medical City, Riyadh (KAMC) involving patients with psoriasis diagnosed in the BESTCare (Riyadh, Saudi Arabia) system between 2015 and 2023.

Selection criteria

All subjects aged 18 or older with a confirmed diagnosis of psoriasis by a dermatologist were included in this study. The study excluded subjects who were under 18 years old, pregnant or lactating, had a thyroid condition that predated psoriasis by more than 90 days, or had thyroid surgery, neck radiation, or medicines that might affect thyroid function.

Sample size

The study included 119 patients with psoriasis who met our inclusion and exclusion criteria.

Data collection and analysis

After receiving IRB approval from King Abdullah International Research Center (KAIMRC), the study group obtained the data of all patients with psoriasis diagnoses from BESTCare (Riyadh, Saudi Arabia). The initial site of involvement was collected on a Microsoft Excel sheet (Redmond, USA). From the medical records, thyroid function tests such as thyroid-stimulating hormone (TSH), triiodothyronine (FT3), thyroxine (FT4), and anti-thyroglobulin antibodies (ANTI-TG) were collected. The normal thyroid function ranges are TSH 0.35-4.94 mIU/L, FT3, 2.90-4.90 mIU/L, and FT4, 9-19 mIU/L. Hyperthyroidism was defined as elevated FT3 and FT4 with decreased TSH. Subclinical hyperthyroidism was defined as normal FT3 and FT4 with decreased TSH. Hypothyroidism was defined as decreased FT3 and FT4 with elevated TSH. Subclinical hypothyroidism was defined as normal FT3 and FT4 with elevated TSH.

Statistical analysis

Qualitative variables were presented as numbers (N) and percentages (%). Quantitative variables were presented as mean and standard deviation. Wilcoxon-Mann-Whitney U test, chi-square test, and Fisher’s exact test were used to look for differences between groups. A p-value less than 0.05 was considered statistically significant. All statistical analyses were performed using SPSS Inc. Released 2003. SPSS for Windows, Version 12.0. Chicago, SPSS Inc.

Ethical consideration

Ethical approval and Institutional Review Board (IRB) approval were granted by King Abdullah International Medical Research Center with the following protocol number: NRC23R/387/06.

## Results

Clinicodemographic characteristics

Table 1 outlines the baseline clinicodemographic data of 119 individuals diagnosed with psoriasis. Among those individuals, 41 (34.4%) were male, while 78 (65.6%) were female. The average age was 44.19 years. Specifically, 54 (45.4%) patients fell below the age of 40, while 65 (54.6%) were aged 40 years or older. The average total duration of illness was 5.35 years. The extremities were the initial site of involvement in the majority of cases (42, 35.3%), followed by the scalp (20, 25.2%) and the trunk (12, 10%). Furthermore, 89 (74.8%) patients exhibited plaque psoriasis, followed by pustular psoriasis (12.6%), erythrodermic psoriasis (4.2%), Palmoplantar psoriasis (3.4%), guttate psoriasis (2.5%), and a small percentage with unknown types (2.5%).

**Table 1:** Clinicodemographic characteristics

Clinical parameters	Frequency
Age (years) (44.19 ± 14.322)	
<40	54 (45.4%)
≥40	65 (54.6%)
Gender	
Male	41 (34.4%)
Female	78 (65.6%)
Total duration of illness (years)	
≤5 years	42 (35.29%)
>5 years	13 (10.92%)
Unknown	64 (53.78%)
Site of Involvement	
Scalp	30 (25.2%)
Extremities	42 (35.3%)
Trunk	12 (10%)
Intergluteal/perianal	0 (0%)
Face	1 (0.8%)
Palm and soles	7 (5.9%)
Axilla/Groin	1 (0.8%)
Unknown	26 (21.8%)
Type of Psoriasis	
Plaque	89 (74.8%)
Palmoplantar	4 (3.4%)
Erythrodermic	5 (4.2%)
Guttate	3 (2.5%)
Pustular	15 (12.6%)
Unknown	3 (2.5%)

Thyroid function tests (TFTs)

Table 2 outlines the summary of thyroid function tests; we found significant deviations from normal levels among patients. Specifically, 9 (7.6%) subjects showed low TSH, while 37 (31.1%) subjects had high TSH levels. For FT3, 11 (9.2%) subjects were low, and 13 (10.9%) subjects were high. Additionally, 9 (7.6%) subjects had low FT4, and 3 (2.5%) subjects had high FT4 levels. Elevated anti-thyroglobulin (anti-TG) and anti-thyroid peroxidase (anti-TPO) antibodies were observed in 15 (12.6%) and 19 (16.0%) of the subjects, respectively. Overall, while 75 (63%) subjects had normal thyroid function, 46 (38.7%) subjects exhibited abnormalities, necessitating further investigation and management.

**Table 2:** Summary of thyroid function tests TSH: Thyroid-stimulating hormone, WNL: Within normal limits, FT3: Free triiodothyronine, FT4: Free thyroxine, Anti-TG: Antithyroglobulin, Anti-TPO=Thyroid peroxidase antibody, TFT: Thyroid function test

Thyroid Function Test	Frequency
TSH	
Low	9 (7.6%)
WNL	73 (61.3%)
High	37 (31.1%)
FT3	
Low	11 (9.2%)
WNL	95 (79.8%)
High	13 (10.9%)
FT4	
Low	9 (7.6%)
WNL	107 (89.9%)
High	3 (2.5%)
Anti-TG	
WNL	104 (87.4%)
High	15 (12.6%)
Anti-TPO	
WNL	100 (84.0%)
High	19 (16.0 %)
TFT	
Normal	75 (63%)
Subclinical hypothyroid	9 (7.6%)
Hypothyroid	33 (27.7%)
Subclinical hyperthyroid	0 (0%)
Hyperthyroid	2 (1.7%)
TFT Impression	
Normal	71 (59.7%)
Abnormal	46 (38.7%)

Various variables associated with abnormal TFTs

Table 3 presents the analysis of various variables to find the association with abnormal TFTs. Analysis of age distribution revealed a slightly higher mean age among individuals with abnormal TFT impressions (45.74 years) compared to those with normal impressions (43.14 years), although statistical significance was not attained (p = 0.249). This suggests that thyroid-related issues are more common among older ages.

**Table 3:** The analysis of various variables to find the association with abnormal thyroid function tests. TFT: Thyroid function test, TSH: Thyroid-stimulating hormone, FT3: Free triiodothyronine, FT4: Free thyroxine, Anti-TG: Antithyroglobulin, Anti-TPO: Thyroid peroxidase antibody, WNL: Within normal limits

Parameters	TFT impression		p-value
	Normal 71 (59.7%)	Abnormal 46 (38.7)	
Age (years)	43.14 ± 15.18	45.74 ± 13.063	0.249a
Age			
≤40 years	36 (50.7%)	17 (36.9%)	0.102b
>40 years	35 (49.3%)	29 (63.1%)	
Gender			
Male	27 (38%)	13 (28.3%)	0.210 c
Female	44 (62%)	32 (71.7%)	
Total duration of illness (Years)	7.63 ± 7.66	6.17 ± 7.24	0.090a
Total duration of illness			
≤5 years	24 (33.8%)	20 (43.5%)	0.414b
>5 years	35 (49.3%)	17 (36.9%)	
Unknown	12 (16.9%)	9 (19.6%)	
Types of psoriasis			
Plaque	52 (73.2%)	35 (76.1%)	0.159 c
Palmoplantar	1 (1.4%)	3 (6.5%)	
Erythrodermic	3 (4.2%)	2 (4.3%)	
Guttate	2 (2.8%)	1 (2.2%)	
Pustular	10 (14.1%)	5 (10.8%)	
Unknown	3 (4.2%)	0 (0%)	
TSH	4.38 ± 18.83	10.75 ± 26.82	0.000a
FT3	3.90 ± 0.94	3.82 ± 1.24	0.995a
FT4	11.57 ± 2.77	12.32 ± 3.18	0.811a
ANTI-TG	90.48 ± 539.241	168.13 ± 491.18	0.301a
Anti-TPO	59.76 ± 235.86	116.17 ± 283.29	0.053a
TSH			0.000 c
Low	3 (4.2%)	6 (13.0%)	
WNL	61 (85.9%)	11 (23.9%)	
High	7 (9.9%)	29 (63.0%)	
FT3			0.238 c
Low	7 (9.9%)	4 (8.7%)	
WNL	58 (81.7%)	35 (76.1%)	
High	6 (8.5%)	7 (15.2%)	
FT4			0.182 c
Low	6 (8.5%)	2 (4.3%)	
WNL	64 (90.1%)	42 (91.3%)	
High	1 (1.4%)	2 (4.3%)	
ANTI-TG			
WNL	63 (88.7%)	39 (84.8%)	
High	8 (11.3%)	7 (15.2%)	
Anti-TPO			0.061 c
WNL	63 (88.7%)	35 (76.1%)	
High	8 (11.3%)	11(23.9%)	
Thyroid Diagnosis			
Normal	66 (93.0%)	8 (17.4%)	0.000 c
Subclinical hypothyroid	1 (1.4%)	8 (17.4%)	
Hypothyroid	3 (4.2%)	29 (63.0%)	
Subclinical hyperthyroid	0 (0%)	0 (0%)	
Hyperthyroid	1 (1.4%)	2 (4.3%)

Despite being higher in females, the distribution across the groups did not reveal a significant difference (p = 0.210), indicating a similar incidence of abnormal TFT levels in both genders. Psoriasis categories did not significantly differ in distribution between those with normal and abnormal TFT levels (p = 0.159), indicating that the type of psoriasis may not have an effect on thyroid function. Significant variations in thyroid hormone levels were discovered, with higher mean TSH levels linked to abnormal TFT levels (p = 0.000), suggesting a possible connection between thyroid malfunction and changed TSH levels. On the other hand, FT3 and FT4 levels did not significantly differ between the groups (p = 0.995 and p = 0.811, respectively). This suggests that although TSH levels in psoriasis patients may indicate thyroid dysfunction, FT3 and FT4 levels may not show the same link.

## Discussion

Medical research continues to delve into the pathogenesis and treatment of psoriasis. Although the association between psoriasis and thyroid dysfunction remains unclear, our study aimed to scrutinize and analyze the correlation between psoriasis and thyroid dysfunction in 119 psoriasis patients.

The majority of our research participants were females (65.6%), while males represented 34.4%. This finding is consistent with a cross-sectional study conducted in Saudi Arabia, which reported a higher prevalence of psoriasis among females [2]. However, another study found a higher prevalence among males in Saudi Arabia [2]. On a global scale, a European study showed a similar prevalence of the disease between males and females, highlighting regional variations in gender distribution [9].

Most patients in our study were 40 years old and above, having a percentage of 54.6%, while the population younger than 40 had a lower percentage of 45.4%. These findings contradict those of Bayomy et al., which found that most of the affected patients were 45 years old or younger [10]. Similarly, Yumnam et al. found that 55% of their study group were 40 years of age or younger [5].

In terms of disease duration, 35.29% of patients had a history of psoriasis for five years or less, 10.92% had a history of more than five years, and 53.78% had an unknown duration of illness. These findings are supported by Yumnam et al. [5], who estimated that most of their study group had the disease for five years or less.

In our study, chronic plaque psoriasis had the largest share (74.8%) among the types of psoriasis, which matches the findings of Alzeer, AlOtair, and Aleisa, who found plaque psoriasis to be the most common type in Saudi Arabia [2]. Globally, this also aligns with the observations of Yumnam et al. [5]. Other types of psoriasis in our study included pustular psoriasis (12.6%), erythrodermic psoriasis (4.2%), palmoplantar psoriasis (3.4%), guttate psoriasis (2.5%), and unknown types (2.5%).

Regarding the initial site of involvement, the extremities were the most common (35.3%) in our study, which is not consistent with the literature, where the scalp is often reported as the most common initial site [5]. However, the scalp followed in our study with 25.2%. This discrepancy might be due to different classification methods, as previous studies often separated the upper and lower extremities. The trunk came third with 10%, palms/soles with 5.9%, and face and axilla/groin with 0.8% each. Lastly, unknown or undocumented sites accounted for 21.8%.

Our study detected thyroid abnormalities in 38.7% of participants. Elevated TSH levels accounted for 31.1%, low TSH levels for 7.6%, and 61.3% of TSH impressions were within normal limits. For FT3, 9.2% of patients had low levels, 10.9% had high levels, and 79.8% were within normal limits. Additionally, 7.6% of patients had low FT4 levels, 2.5% had high FT4 levels, and 89.9% were within normal limits. Elevated anti-thyroglobulin (Anti-TG) was observed in 12.6% of patients, while 87.4% were within normal limits. Elevated anti-thyroperoxidase (Anti-TPO) was noticed in 16% of patients, with 84% within normal limits. Hypothyroidism was diagnosed in 27.7% of patients, similar to the findings by Yumnam et al. [5]. Additionally, 7.6% of patients were diagnosed with subclinical hypothyroidism, 1.7% with hyperthyroidism, and no cases of subclinical hyperthyroidism were observed. The majority of patients had never been diagnosed with any thyroid disease, accounting for 63% of the participants. The final TFT impressions showed that 59.7% of participants had normal findings, while 38.7% had abnormalities. In comparison, Yumnam et al. detected 19.8% of abnormal thyroid findings among their study group [5].

Our study investigated the association between psoriasis and thyroid abnormalities and found no direct correlation between specific types of psoriasis and the likelihood of thyroid issues. However, it is critical to note that further research is necessary to comprehensively understand the relationship between psoriasis and thyroid diseases. These studies should also consider other factors that contribute to both conditions, such as diabetes and obesity, which have been suggested to exacerbate both psoriasis and thyroid dysfunction [11-12]. By taking these additional factors into account, we can gain a more comprehensive understanding of the connection between psoriasis and thyroid abnormalities.

Limitations

The data available for psoriasis patients were limited, with most patients not having prior screening for other autoimmune diseases. There was a lack of follow-up visits; thus, the description of psoriasis lesions, the chronicity of the disease, and the course of either the psoriasis or the thyroid dysfunction could not be obtained. Lastly, this was a hospital-based study, which limited the number of participants, making it a small study group.

## Conclusions

This study investigated the relationship between psoriasis and thyroid dysfunction in a sample of 119 patients, revealing no direct correlation between specific types of psoriasis and the likelihood of thyroid abnormalities. While these results suggest that psoriasis itself may not serve as a direct predictor for thyroid dysfunction, the relationship between these two conditions remains complex and multifactorial. Further research is needed to explore potential mechanisms, particularly how other factors, such as metabolic disorders like diabetes, obesity, and environmental influences, might exacerbate both psoriasis and thyroid disease.

Although the current study is limited by the relatively small sample size and the lack of long-term follow-up, its findings underscore the necessity of a comprehensive understanding of the interplay between psoriasis and its comorbidities. These insights are essential for refining treatment strategies that address dermatological manifestations and the broader systemic issues that may impact patients. Future studies should aim to include larger multi-center studies and longitudinal follow-ups to strengthen our understanding of the potential link between psoriasis and thyroid dysfunction and to develop more effective management approaches for those affected.
